# Медиакальциноз у пациентов с сахарным диабетом: этиопатогенетические, патофизиологические гистопатологические аспекты

**DOI:** 10.14341/probl13360

**Published:** 2024-04-24

**Authors:** О. Н. Бондаренко, М. В. Ярославцева, Г. Р. Галстян, Н. Г. Мокрышева

**Affiliations:** Национальный медицинский исследовательский центр эндокринологии; Национальный медицинский исследовательский центр эндокринологии; Национальный медицинский исследовательский центр эндокринологии; Национальный медицинский исследовательский центр эндокринологии

**Keywords:** медиакальциноз, сахарный диабет, этиопатогенез, гистопатология медиакальциноза, гладомышечные клетки сосудов

## Abstract

В обзоре обобщены результаты зарубежных и отечественных исследований, посвященных механизмам развития и патогенезу кальцификации сосудов (КС). Рассматриваются этиопатогенетические, патофизиологические и гистоморфологические особенности медиакальциноза, определяющие изменения сосудистого русла у больных сахарным диабетом (СД). Обозначена роль таких факторов риска заболевания, как повышенный уровень гликемии, изменение уровня инсулина, нарушение обмена липидов, ожирение, артериальная гипертензия, ХБП и старение. Обсуждается роль прокальцифицирующих и антикальцифицирующих факторов ремоделирования сосудистой стенки. Выявление информативных молекулярных маркеров и факторов КС  позволит в  перспективе разработать эффективные стратегии медикаментозного управления риском их прогрессирования и  индивидуальные программы профилактики для повышения качества и продолжительности жизни у пациентов с сердечно-сосудистыми заболеваниями.

## Abstract (en)

In a review of the generalized results of foreign and domestic studies on the development mechanism and pathogenesis of vascular calcification. The etiopathogenetic, pathophysiological and histomorphological features of mediacalcinosis, which reveal changes in the vascular bed in patients with DM, are considered. The role of risk factors for diseases, such as increased glycemia, changes in insulin levels, impaired lipid metabolism, obesity, arterial hypertension, CKD and aging, is indicated. The role of procalcifying and anticalcifying factors in colonic vascular remodeling is discussed. Identification of informative molecular markers and factors of CS will allow in the future to develop effective strategies for drug management of the risk of their progression and individual prevention programs to improve the quality and life expectancy in patients with cardiovascular diseases.

## ВВЕДЕНИЕ

Медиакальциноз, или склероз Менкеберга, является распространенным осложнением сахарного диабета (СД) и одной из важнейших причин неблагоприятных сердечно-сосудистых исходов, ампутаций и смерти. Медиакальциноз определяется как хроническое системное сосудистое заболевание, отличное от атеросклероза, ассоциированное с СД, старением и хронической болезнью почек (ХБП). Механизмы, лежащие в основе развития медиакальциноза у больных СД, не до конца изучены. Тем не менее имеющиеся на сегодняшний день фундаментальные научные и клинические данные об особенностях кальцификации сосудов (КС) позволяют дифференцировать поражение артерий у пациентов с СД и у больных с классическим атеросклерозом, развивающимся в общей популяции. Отличительные признаки заболевания включают диссеминированное и прогрессирующее отложение фосфата кальция в медиальном слое артерии, длительное бессимптомное течение, а также нарушение гемодинамики, тесно связанное с хронической ишемией, угрожающей потерей конечности. Данное патологическое состояние включает накопление кристаллов гидроксиапатита в среднем слое артериальной стенки, что приводит к ее прогрессирующему обызвествлению. При определенных условиях возможна трансформация гладкомышечных клеток (ГМК) сосудистой стенки артерий из их первоначального сократительного фенотипа в остеобластоподобный. Обнаружение костного морфогенетического белка в образцах кальцинированных атеросклеротических бляшек (АСБ) человека положило начало биологической теории сосудистой кальцификации. Были сформулированы основные про- и антикальцифицирующие факторы ремоделирования сосудистой стенки, определен ряд клинических состояний, влияющих на развитие КС. Целью данного обзора является обобщение имеющихся данных в литературе эпидемиологических, этиопатогенетических, патофизиологических и гистопатологических аспектов медиакальциноза у пациентов с СД. Понимание механизмов развития и знание ключевых факторов риска медиакальциноза позволит в будущем создать эффективные меры профилактики и патогенетически обоснованные подходы к лечению данной патологии.

## ЭПИДЕМИОЛОГИЯ МЕДИАКАЛЬЦИНОЗА

Истинная распространенность медиакальциноза неизвестна. Зачастую данное морфологическое поражение сосудов представляет собой случайную находку или ошибочно трактуется как атеросклероз, поэтому может вовремя не распознаваться. Диагностика медиакальциноза артерий нижних конечностей пациентов с подозрением на заболевание артерий нижних конечностей (ЗАНК), оцениваемая с помощью лодыжечно-плечевого индекса >1,3, составляет около 0,5% среди взрослых, при соотношении мужчин и женщин 3:2 [1]. Тем не менее значение лодыжечно-плечевого индекса >1,3 является недостаточно точным параметром для постановки диагноза [2]. По данным исследований, распространенность медиакальциноза была выявлена у 17–42% пациентов с СД 2 типа (СД2) [3], у 27–40% пациентов с прогрессирующей ХБП [4] и у 72% пациентов с хронической ишемией, угрожающей потерей конечности [5].

Известно, что около одной трети пациентов, направленных на инвазивное лечение ишемической болезни сердца (ИБС), имеют выраженный кальциноз коронарных артерий [6]. Установлено, что кальцификация артерий сердца чаще выявляется при распространенном многососудистом поражении в старшей возрастной группе, в особенности при сочетанных поражениях в других сосудистых бассейнах, то есть рассматривается как маркер более тяжелого, генерализованного атеросклеротического процесса. Анализ доступной литературы показывает, что минерализация аорты и артерий — достаточно частое явление, причем во всех исследованиях главная роль в распространении данного процесса отводится возрасту больных, по мере увеличения которого нарастают как распространенность, так и выраженность кальцификации артериальной стенки. У пациентов старше 50 лет кальциноз встречается в 16% случаев у женщин и в 20% случаев у мужчин. Между тем в возрасте старше 70 лет частота его увеличивается до 93 и 98% у женщин и мужчин соответственно [7].

## ЭТИОПАТОГЕНЕТИЧЕСКИЕ АСПЕКТЫ МЕДИАКАЛЬЦИНОЗА

В настоящее время установлено, что медиакальциноз ассоциирован с СД, старением и повышенным риском развития хронической почечной недостаточности (ХПН), заболеванием коронарных артерий, ампутациями, высокой смертностью у больных СД [8]. Кальцификация средней оболочки артерий происходит независимо от атеросклеротического процесса, хотя и может сочетаться с ним [9]. В практической деятельности рассматриваются четыре основных вида кальцификации сосудистой стенки: кальциноз интимы, медиакальциноз, кальцификация клапанов сердца, кальцийфилаксия [10]. Наиболее важное с клинической точки зрения значение на сегодняшний день имеют два варианта кальциноза — медиакальциноз и кальциноз интимы [11]. В публикации Менкеберга указывалось, что структурные изменения были наиболее выражены в дистальных отделах сосудистого русла — артериях голеней и стоп. Считается, что выраженная степень склероза Менкеберга наблюдается при тяжелых метаболических и электролитных нарушениях, сопровождающих некоторые заболевания (СД, ХПН, гипервитаминоз D, остеопороз, прием варфарина, дефицит витамина К, ревматоидный артрит и некоторые другие), менопаузу и старение организма [7] (рис. 1, 2).

**Figure:**
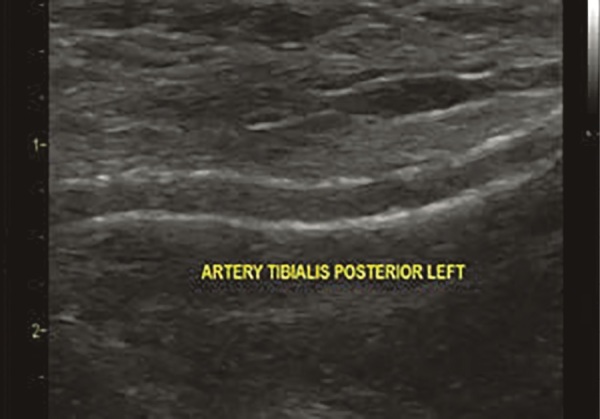
Рисунок 1. Эхограмма. В-режим. Кальциноз стенки задней большеберцовой артерии у пациента 73 лет с СД2 более 20 лет и хронической почечной недостаточностью.

**Figure:**
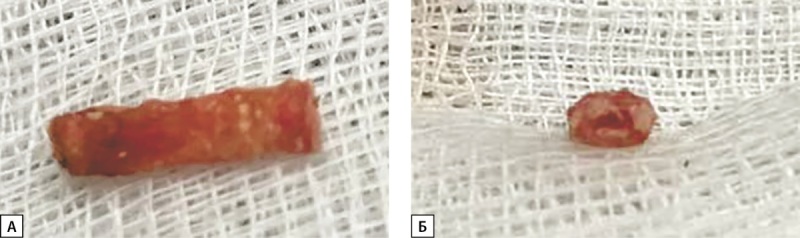
Рисунок 2. Фотографии. Кальцинированная тыльная плюсневая артерия стопы в 1 межфаланговом промежутке (операционный материал).А — продольный срез, Б — поперечный срез.

Пусковые механизмы развития медиакальциноза преобладают в зависимости от патологических изменений при том или ином виде заболевания. Так, например, при ХПН, особенно у пациентов, находящихся на заместительной почечной терапии, триггером является нарушение фосфорно-кальциевого обмена [7]. У пациентов с СД ключевая роль в патогенезе кальцификации медии отводится декомпенсации углеводного обмена, для которой характерна активация полиолового пути, приводящего к окислительному стрессу, индукция воспалительного ответа конечными продуктами гликирования, что способствует трансформации ГМК.

В исследовании Edmonds М. [12] и соавт. [13][14] у пациентов с СД была отмечена сильная корреляция между КС, вибрационной чувствительностью и уровнем креатинина сыворотки крови [15], что впервые продемонстрировало взаимосвязь развития склероза Менкеберга с такими осложнениями СД, как диабетическая полинейропатия и ХПН. Впервые медиакальциноз артерий по данным рентгенологического исследования был верифицирован у пациентов с диабетической нейроостеоартропатией в 90% [16] и 78% случаев [17] соответственно. В дальнейшем аналогичные рентгенологические признаки КС, характеризующиеся обызвествлением стенок артерий, были выявлены у пациентов с нейропатической формой синдрома диабетической стопы [14], (рис. 3).

**Figure:**
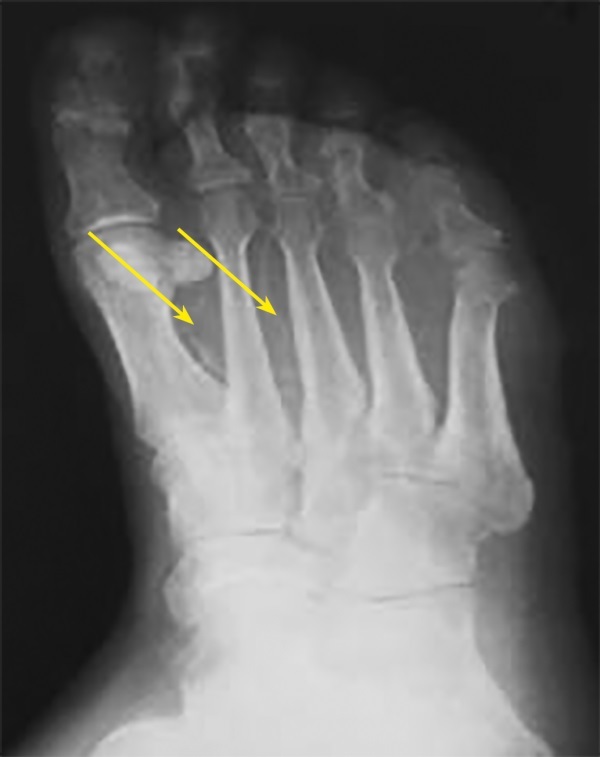
Рисунок 3. Рентгенограмма стопы. Кальциноз артерий стопы (указано стрелками).

Forst c соавт. [17] обнаружили достоверную связь между медиакальцинозом и автономной нейропатией, выявляемой с помощью кардиоваскулярных тестов и оценки функции потоотделения. Однако следует учитывать, у пациентов с медиакальцинозом необходимо с большой осторожностью интерпретировать результаты тестов на выявление автономной нейропатии, поскольку нарушение вазоконстрикции может быть следствием структурных изменений сосудистой стенки, а не признаком симпатической деиннервации. Кроме того, клинические признаки кальциноза артерий затрудняют диагностику автономной дисфункции. В работе Gentile и соавт. [18] медиакальциноз периферических артерий отмечен у 37 из 41 пациента с автономной нейропатией, которая отсутствовала в контрольной группе без вегетативной нейропатии (р<0,001). Медиальная артериальная кальцификация была описана после поясничной симпатэктомии у пациента с редким наследственным заболеванием — транстиретиновой семейной амилоидной полинейропатией, в основе которой лежит отложение амилоида в периферических нервах, что ведет к аксональной дегенерации [18]. В исследовании Goebel FD и соавт. также было показано, что односторонняя симпатэктомия значительно чаще приводит к КС нижних конечностей в сравнении с другой нижней конечностью — 89 против 18% (р<0,01) [19]. В экспериментальных исследованиях на животных односторонняя симпатэктомия приводила к прогрессированию атеросклероза на оперированной стороне [20]. Таким образом, важная роль в патогенезе медиакальциноза у больных с диабетической макроангиопатией отводится автономной нейропатии. Вовлеченность нервной системы в развитие и прогрессирование медиакальциноза может являться объяснением дистального характера поражения артерий, как при СД, так и при других патологиях, осложненных дистальной нейропатией.

## ПАТОФИЗИОЛОГИЧЕСКИЕ МЕХАНИЗМЫ РАЗВИТИЯ МЕДИАКАЛЬЦИНОЗА

В последние годы КС была идентифицирована как сложно регулируемый патофизиологический процесс, опосредованный различными клетками, который характеризуется трансформацией гладкомышечных клеток сосудов (ГМКС) из их первоначального сократительного фенотипа в остеобластоподобный фенотип после стимуляции различными факторами. ГМКС являются первичными клетками, ответственными за КС. Продукты деградации ГМКС после апоптоза или некроза (апоптозные тела и матричные везикулы соответственно) участвуют в инициации кальцификации [21]. Гипергликемия и окислительный стресс ускоряют формирование конечных продуктов гликирования (КПГ), способствуя дифференцировке ГМКС в остеобластоподобные клетки. Ключевым моментом в инициации процессов КС признается формирование остеогенного фенотипа клеток-мишеней. Последние экспрессируют широкий спектр регуляторных протеинов и сигнальных молекул (костный морфогенетический белок-2 (BMP-2 — bone morphogenetic protein 2), щелочную фосфатазу (ALP — alkaline phosphatase), белок-транскрипционного фактора Cbfa1 (core-binding factor α1), известного также как внутриядерный фактор транскрипции (RUNX2 — runt related transcription factor 2), остеокальцин или костный Gla белок (OCN-Osteocalcin или BGP — Bone Gla protein), коллаген I типа (Col I — Collagen-1). Остеогенно трансформированные клетки-мишени продуцируют гидроксиапатит. Депозиты кальция откладываются в средней оболочке артерий в случае развития склероза Менкеберга [22].

Кроме того, кальцификации способствует снижение экспрессии ингибиторов КС-матриксного Gla белка (MGP — matrix gla protein), неорганического пирофосфата, остеопротегерина (OPG — osteoprotegerin), остеопонтина (OPN — osteopontin), фетуина А (FA — fetuin A) — с одной стороны.

С другой стороны, хроническая гипергликемия и окислительный стресс с образованием активных форм кислорода, повышение активности воспалительных цитокинов, повышение КПГ (AGEs) и рецепторов (RAGE), которые синтезируются остеобластами и остеокластами и составляют сигнальный путь AGE/RAGE, и других факторов, стимулируют процесс кальцификации [23].

Таким образом, на сегодняшний день выделены наиболее чувствительные и специфические биологические маркеры, играющие важную роль в ремоделировании сосудистой, а также костной ткани [24]. Основными из них являются липиды, неорганический фосфат и пирофосфат (Pi/PPi), сигнальные пути Wnt, специфические транскрипционные факторы (Cbfa1/Runx2, Msx2, Sox9), система RANK/RANKL/OPG, фактор роста фибробластов-23 (FGF-23)/белок Klotho, неколлагеновые белки (морфогенетические белки BMP-2,4,7), остеопонтин (OPN), матриксный Gla протеин (MGP), остеокальцин (OCN), остеонектин (ON), Fetuin-A) и т.д. (таблица 1). В результате системного и локального дисбаланса между ингибиторами и активаторами кальцификации происходит фенотипическая трансформация ГМКС в клетки с остеогенным потенциалом.

**Table:** Таблица 1. Прокальцифицирующие и антикальцифицирующие факторы ремоделирования сосудистой стенки

Прокальцифицирующие факторы	Антикальцифицирующие факторы
Фосфат	Пирофосфат
Щелочная фосфатаза	BMP — 7
BMP — 2, 4	Fetuin A
Cbfal/Runx2	FGF-23/Klotho
OCN	OPN
RANKL	MGP
Msx2	Магний
Sox9	Витамин К
Провоспалительные факторы	ЛПВП
Варфарин	Эстрогены
ЛПНП	Адипонектин
Кальций	Инсулиноподобный фактор роста 1
Уремические токсины	
Дефицит витамина D	
Лептин	

## ФАКТОРЫ РИСКА СОСУДИСТОЙ КАЛЬЦИФИКАЦИИ

## Повышенный уровень гликемии

Достаточное количество исследований посвящено механизмам патогенеза КС вследствие хронической гипергликемии. Установлено, что in vitro ГМКС, культивируемые в условиях повышенного уровня глюкозы, экспрессируют RANX2, BMP-2 и остеокальцин. В работе, проведенной на мышиных ГМК, высокий уровень глюкозы способствовал их превращению в остеобласты с высокой экспрессией декорина [25].

Основная функция декорина включает регуляцию во время клеточного цикла. Экзогенный декорин ингибирует активность ферментов, разрушающих внеклеточный матрикс и деградацию коллагена, одновременно активируя отложение матрикса и усиливая кальцификацию. Аналогично в исследованиях in vitro было показано, что высокий уровень глюкозы вызывает кальцификацию ГМКС [26].

При воздействии окислительного стресса на ГМКС в условиях повышенного уровня глюкозы и других стимулирующих факторов они дифференцируются в остеобластоподобные клетки и секретируют большое количество белков костной ткани. R. Kawakami и соавт. [27] обнаружили, что семейство белков S100 у больных СД тесно связано с КС.

Гипергликемия способствует увеличению секреции S100A9 и повышению экспрессии белковых рецепторов RAGE. В среде с высоким содержанием глюкозы провоспалительные макрофаги высвобождают кальцинированные внеклеточные везикулы, которые формируют атеросклеротические микрокальцификаты через ось S100A9-RAGE [26].

Глюкозозависимая продукция митохондриального супероксида становится чрезмерной, что приводит к активации NF-κB [26]. Ядерный фактор NF-κB представляет значимый транскрипционный фактор, активирующий многие провоспалительные механизмы в развитии атеросклероза и медиакальциноза. RAGE, рецептор к конечным продуктам гликирования, при взаимодействии с белками группы S100A запускает каскад реакций, который способствует увеличению транскрипции ядерного фактора NF-κB и повышению уровня активных форм кислорода, что ведет к развитию локального оксидативного стресса. Связывание RAGE с лигандом стимулирует продукцию основных провоспалительных цитокинов — IL-6, IL-1β, TNF-α. Эти маркеры также стимулируют локальное воспаление в нескольких типах клеток: эндотелиальных, гладкомышечных клетках сосудистой стенки, лейкоцитах. S100A9 повышает секрецию провоспалительных цитокинов с помощью таких механизмов, как образование активных форм кислорода и активация чувствительных к ним транскрипционных факторов (NF-κB). Гипергликемия способствует также повышенной экспрессии белков BMP -2/4. У больных СД повышена экспрессия BMP -2/4 в аорте. Этот белок способствует остеогенной дифференцировке ГМКС, что приводит к их кальцификации.

## Нарушение обмена липидов

Питание с повышенным содержанием жиров, особенно животного происхождения, способствует развитию ожирения, артериальной гипертензии и СД. У пациентов с СД окисленные липопротеины низкой плотности (ЛПНП) вносят вклад в прогрессирование атеросклероза и кальцификацию средней оболочки артерии (медии). Крупное обсервационное исследование пациентов с СД1 в шведской клинической практике показало, что каждое повышение уровня холестерина ЛПНП на 1 ммоль/л приводит к повышению риска сердечно-сосудистых заболеваний (ССЗ) на 9% у лиц, не получавших терапию статинами. В исследовании был сделан вывод о том, что холестерин ЛПНП, по-видимому, не является достаточно точным маркером сердечно-сосудистого риска при первичной профилактике у пациентов с СД1 [28]. Европейское исследование осложнений инсулинозависимого СД (EURODIAB IDDM Complications Study) также показало, что холестерин ЛПНП не является предиктором сердечно-сосудистых заболеваний [29]. Тем не менее стандарты медицинской помощи Американской ассоциации диабета (ADA — American Diabetes Association) предполагают, что уровень холестерина ЛПНП составляет 2,6 ммоль/л и более является маркером повышенного сердечно-сосудистого риска [30]. Исследования in vitro показали, что липопротеины высокой плотности (ЛПВП) не подвергаются окислению и оказывают протективное действие на артериальную стенку благодаря ингибирующему действию на КС [26]. Гипергликемия, по-видимому, оказывает более существенное влияние на сердечно-сосудистые риски при СД1, чем при СД2. В моделях прогнозирования рисков для пациентов с СД1 общий холестерин и холестерин ЛПВП более важны, чем холестерин ЛПНП в оценке прогноза неблагоприятных сердечно-сосудистых исходов [31]. Важно отметить, что кажущиеся нормальными концентрации холестерина в сыворотке крови, часто наблюдаемые при СД1, скрывают атерогенный липидный профиль с повышенным содержанием липопротеинов промежуточной (средней) плотности ЛППП и ЛПНП, а также дисфункциональных ЛПВП [32]. Повышенный уровень аполипопротеина А-1 (апо А1), содержащегося в ЛПВП, является независимым генетически детерминированным причинным фактором риска развития сердечно-сосудистых заболеваний [33]. Влияние СД1 на уровень апо А1 неясно, но проспективное обсервационное исследование показало, что его уровень, превышающий 30 мг/дл, может иметь значение для прогнозирования сердечно-сосудистых событий у пациентов с СД1 [34]. Интересно, что интенсивное лечение в исследовании DCCT (Diabetes Control and Complications Trial) было связано со снижением апо А1, а также апо В [35]. Снижение уровня ЛПВП часто сопровождается повышением уровня лептина в кровообращении. Высокий уровень лептина, в свою очередь, активирует остеогенный сигнальный путь BMP, стимулируя дифференцировку ГМКС в остеобласты и опосредует КС [36]. Размер частиц ЛПНП, их гликирование и окисление связаны с высоким риском ССЗ. Липопротеины очень низкой плотности (ЛПОНП) легче проникают в стенку артерии, чем ЛПНП, более восприимчивы к окислительному стрессу, имеют длительный период полувыведения из плазмы и обладают сниженным сродством к связыванию с рецепторами ЛПНП [26]. Proudfood D. и соавт. [37] отметили, что при развитии дислипидемии ацетилированные ЛПНП увеличивают остеогенный фенотип ГМКС в 3 раза. Таким образом, ЛПНП индуцируют их дифференцировку и повышают активность щелочной фосфатазы. Напротив, ЛПВП ингибируют путь остеогенной дифференцировки ГМКС [28]. Кроме того, гиперхолестеринемия усиливает окислительный стресс и ускоряет КС, индуцированную витамином Д [38]. Bjornstad Р. и соавт. [39] установили, что гипертриглицеридемия представляет независимый прогностический фактор прогрессирования кальцификации коронарных артерий у пациентов с СД. Некоторые исследования показали, что гиперлипидемия связана с нарушением проведения сигналов на одном из его этапов по пути Wnt/β-катенин, который играет важную роль в КС [40].

## Изменение уровня инсулина

В физиологических условиях инсулин оказывает ангиопротективное действие при развитии кальциноза. Многочисленные исследования показали, что оксид азота (NO) ингибирует активацию тромбоцитов и ограничивает миграцию и пролиферацию ГМКС [41]. Инсулин стимулирует эндотелий сосудов к высвобождению NO, который впоследствии окисляет липопротеины, тем самым снижая скорость кальцификации интимы и препятствуя остеогенной дифференцировке ГМКС [42].

Повышенная секреция инсулина ассоциирована с инсулинорезистентностью (ИР). Инсулинорезистентность, по-видимому, предсказывает степень развития КС и может быть связана с более высоким риском сердечно-сосудистых заболеваний, особенно у пациентов с СД. В случае инсулинорезистентности в печень поступает большое количество свободных жирных кислот. При этом организм компенсирует это состояние избыточным поглощением триглицеридов печенью, а также образованием и секрецией ЛПОНП, что сопровождается повышением риска КС. Как показано в исследовании Т. Iguchi и соавт., пациенты с более высоким уровнем ИР склонны к массивным фиброзным атеросклеротическим наслоениям в сосудах тонкой кишки [43]. ИР способствует развитию атеросклероза, индуцируя воспалительную активность кровеносных сосудов и иммунных клеток. Как было показано Queralt Martín-Saladich и соавт., чем выше индекс ИР, тем выше кальцификация коронарных артерий [44]. В другом исследовании, проведенном с участием 1632 пациентов без СД, было показано, что ИР связана только с кальцинозом коронарных артерий, но не аорты [45].

Клетки Купфера являются резидентными макрофагами печени и играют важную роль в поддержании функции печени [41]. В случае гиперинсулинемии клиренс клеток Купфера в печени уменьшается [46]. Это изменение приводит к снижению клиренса липополисахаридов, абсорбирующихся из ЖКТ, и соответствующему увеличению циркулирующих уровней липополисахаридов и инсулина. Кроме того, в исследовании на крысах ингибирование клеток Купфера вызывало повышение уровня глюкозы в организме и стимулировало чрезмерную секрецию инсулина, приводя к ИР [47]. Stefan N. и соавт. [48] выявили, что плазменный глобулин А ингибирует передачу сигнала инсулина и индуцирует ИР, что приводит к атеросклерозу.

## Ожирение

Ожирение является независимым фактором риска развития сердечно-сосудистых заболеваний. Наиболее часто избыточный вес встречается среди пациентов с СД2, в то время как распространенность ожирения у пациентов с СД1 не выше, чем у населения в целом. У пациентов с СД1 ожирение ассоциировано с наличием и прогрессированием кальциноза коронарных артерий, между тем связь между ожирением и СД2 в настоящее время не до конца изучена. В Британском исследовании было показано, что у пациентов с СД2 смертность от всех причин была выше у пациентов с индексом массы тела (ИМТ) 35–54 кг/м² и 20–24 кг/м² [53]. В недавнем отечественном исследовании была продемонстрирована высокая распространенность ишемической болезни сердца у пациентов с висцеральным ожирением и кальцификацией коронарных артерий [49]. Вместе с этим, по данным ряда исследований, выявлена взаимосвязь между объемом эпикардиальной жировой ткани, являющейся гормонально-активным образованием и кальцинозом коронарных артерий. Взаимодействие между эпикардиальной жировой тканью и коронарными артериями происходит через паракринную активность, включающую маркеры воспаления, которые могут опосредовать и индуцировать кальцификацию [50]. В исследовании, в котором приняли участие 1414 афроамериканцев, кальциноз коронарных артерий и кальциноз абдоминальной аорты были ассоциированы с эпикардиальным жиром [51].

## Артериальная гипертензия

Ренин-ангиотензин-альдостероновая система (РААС) является основным патогенетическим фактором, способствующим апоптозу, росту и дифференцировке ГМКС, что позволяет предположить возможное участие этой системы в КС. Ангиотензин II способствует дифференцировке ГМКС в остеобласты путем активации RANKL [52]. Аналогичным путем повышение активности альдостерона сопровождается повышением артериального давления и прогрессированием сердечно-сосудистых заболеваний. При повышении уровня альдостерона в крови активируются факторы воспаления и пролиферации, негативно влияя на состояние артерий.

Исследования также показали, что промоторная последовательность гена pit-1, который является гипофизарно-специфическим фактором транскрипции, может содержать элементы распознавания микроРНК на своих целевых мессенджерных РНК. МикроРНК, которые представляют собой очень короткие некодирующие РНК, играют важную роль в регуляции экспрессии генов и прогрессировании заболевания. В данном случае речь идет о влиянии повышенного уровня альдостерона на остеоиндукцию через pit-1. Pit-1 регулирует поглощение фосфата и необходим для фенотипической трансформации и кальцификации ГМКС [25]. X. Li и соавт. [53] установили, что подавление pit-1 малых интерферирующих РНК снижало уровни матричной РНК Cbfa1 (семейство транскрипционных факторов, известных также под названиями Runx2 или Osf-2, osteoblast specific transcription factor 2, остеобласт-специфический фактор транскрипции 2) и ЩФ и ингибировало кальцификацию сосудов.

Таким образом, дисрегуляция РААС тесно связана с КС. Это делает РААС привлекательной терапевтической мишенью для разработки эффективных методов фармакотерапии медиакальциноза при сердечно-сосудистых заболеваниях.

## Заболевания почек

Эпидемиологические исследования показали, что у пациентов с СД вероятность развития ХБП примерно в два раза выше, чем у людей без нарушения углеводного обмена. Исследование M. Wang и соавт. пациентов с терминальной стадией ХПН, получающих терапию программным гемодиализом, показало, что распространенность КС у них составила 77,4%. Частота кальциноза коронарных артерий у недиализных пациентов с СД2 с поражением почек была выше, чем у пациентов с СД2 без повреждения почек (95 и 59%), а медиана оценки коронарного кальция была значительно выше [54]. По мере прогрессирования ХПН возрастает вероятность нарушения метаболизма кальция и фосфора, представляя дополнительный риск кальциноза. Внескелетная кальцификация часто встречается у пациентов с ХБП; ее распространенность увеличивается по мере нарастания ХПН и увеличения продолжительности диализа.

Ключевую роль в развитии КС играет провоспалительное окружение в системном кровообращении, характеризующееся непрерывным повышением содержания воспалительных белков и цитокинов, часто встречающееся у пациентов с хроническим заболеванием почек. Небольшое увеличение этих показателей положительно коррелирует с активаторами кальцификации.

## Процесс старения

Согласно данным литературы, старение способствует КС путем активации ряда процессов: остеогенной трансформации гладкомышечных клеток, высвобождению везикул эндотелиальными клетками, ремоделированию внеклеточного матрикса, дисбалансу метаболизма фосфора, повреждению ДНК, воспалительной реакции и снижению экспрессии антивозрастных факторов [55].

В то же время у пожилых людей усиливается сочетанное воздействие на артериальную стенку других негативных факторов. Так, например, количество КПГ, содержащихся в тканях у пациентов с СД2, по мере старения больше, чем у пациентов без СД [56]. При гипергликемии сигналы AGE/RAGE передаются через PKC (протеинкиназа С, PKC — protein kinase C), p38 MAPK (митоген-активируемые протеинкиназы, p38 — mitogen-activated protein kinases), TGF-β (трансформирующий фактор роста бета, TGFβ1 — transforming growth factor β1) и внутриклеточный сигнальный путь NF-κB (ядерный фактор каппа в, NF-κB — nuclear factor κB), а также другие сигнальные пути, которые увеличивают количество белков костного матрикса [57].

Более того, было показано, что передача сигналов AGE/RAGE увеличивает окислительный стресс и способствует КС, опосредованной СД, путем активации Nox-1 (NADPH-оксидаза (NOX)) и снижения экспрессии гена SOD-1 [58].

## ГИСТОПАТОЛОГИЯ МЕДИАКАЛЬЦИНОЗА

Медиакальциноз распространяется преимущественно на артерии мышечного типа, к которым относятся сосуды среднего и мелкого калибров. В стенках этих артерий имеется относительно большое количество ГМК. Ранняя стадия обызвествления средней оболочки артерий характеризуется появлением кристаллов фосфата кальция в промежуточном веществе около эластических волокон. На поздних стадиях развития медиакальциноза наблюдается формирование массивных прогрессирующих очагов отложения солей кальция в средней оболочке вдоль эластических волокон, что сопровождается фрагментацией внутренней эластической мембраны [59].

Исследования последних лет указывают на сочетанные патологические изменения интимы и медии у пациентов с диабетической макроангиопатией с преимущественным поражением средней оболочки артерий [60]. Оценка образцов гистологических срезов материалов ампутированных конечностей у больных с диабетической нефропатией показала, что неатероматозное интимальное утолщение стенок артерий (отсутствие липидов и макрофагов) само по себе может привести к значительному стенозу, а иногда — к окклюзии или тромбозу [60].

В другом исследовании гистологический анализ операционного материала конечностей у пациентов с СД2 (80%), терминальной стадией ХБП (48,3%) и ЗАНК выявил признаки выраженного (более 50%) циркулярного и полисегментарного кальциноза средней оболочки артерий, а также атеросклеротическое поражение с формированием значимых стенозов [59].

Результаты патоморфологических исследований операционного материала конечностей больных СД и ЗАНК установили тесную связь между выраженной гиперплазией интимы, прогрессирующим медиакальцинозом, распространяющимся на артерии голени и стопы, и развитием тромботических окклюзий [60].

Таким образом, основным элементом диабетической макроангиопатии является поражение средней оболочки артерии — медии мышечных артерий, которое может сопровождаться также патологией интимы.

В целом, полученные на сегодняшний день данные о гистопатологии медиакальциноза свидетельствуют о его значительном вкладе в развитие и прогрессирование ЗАНК у пациентов с СД.

## ЗАКЛЮЧЕНИЕ

Медиакальциноз артерий является распространенным осложнением у пациентов с СД и одной из важнейших причин сердечно-сосудистых заболеваний и смерти. Процессы и механизмы КС являются сложно регулируемым патофизиологическим феноменом, опосредованным различными клетками, который характеризуется трансформацией ГМКС из их первоначального сократительного фенотипа в остеобластоподобный фенотип после стимуляции различными про- и антикальцифицирующими факторами. Важнейшую роль среди факторов риска при СД играют хроническая гипергликемия и оксидативный стресс, а в случае сопутствующей ХПН, особенно у пациентов, находящихся на диализе, пусковым механизмом является нарушение фосфорно-кальциевого обмена. Дополнительными факторами риска развития медиакальциноза являются такие патологические состояния, как гиперинсулинемия и инсулинорезистентность, нарушение обмена липидов, ожирение, дисрегуляция РААС при артериальной гипертензии и старение. В настоящее время формируется понимание многогранности взаимодействия этих патологических процессов, а также их клинико-прогностической роли. Глубокое знание аспектов патогенеза медиакальциноза может лечь в основу разработки мероприятий по его профилактике у пациентов с СД и наметить цели фармакологической терапии у больных с уже развившимся заболеванием.

## ДОПОЛНИТЕЛЬНАЯ ИНФОРМАЦИЯ

Источники финансирования. Работа выполнена по инициативе авторов без привлечения финансирования.

Конфликт интересов. Авторы декларируют отсутствие явных и потенциальных конфликтов интересов, связанных с содержанием настоящей статьи.

Участие авторов. Все авторы одобрили финальную версию статьи перед публикацией, выразили согласие нести ответственность за все аспекты работы, подразумевающую надлежащее изучение и решение вопросов, связанных с точностью или добросовестностью любой части работы.
